# Cytotoxic Effect of Some 1, 4-Dihydropyridine Derivatives Containing Nitroimidazole Moiety

**Published:** 2011

**Authors:** Ramin Miri, Katayoun Javidnia, Zahra Amirghofran, Seyyed Hossein Salimi, Zahra Sabetghadam, Savis Meili, Ahmad Reza Mehdipour

**Affiliations:** a ***Medicinal and Natural Products Chemistry Research Center, Shiraz University of Medical Sciences, Shiraz, Iran.***; b ***Department of Medicinal Chemistry, Faculty of Pharmacy, Shiraz University of Medical Sciences, Iran.***; c ***Department of Immunology, Faculty of Medicine, Shiraz University of Medical Sciences, Shiraz, Iran.***

**Keywords:** 1,4-Dihydropyridines, Cytotoxicity, MTT assay, Nitroimidazole

## Abstract

The 1,4-dihydropyridine (DHP) derivatives are a known class of calcium channel blockers. Some derivatives of DHP showed significant cytotoxicity. It was shown that this effect may not be the result of interaction with Ca^2+^ channels. In this study, we performed an investigation about the intrinsic cytotoxicity of some derivatives of DHP containing nitroimidazole moiety on their C4 position on four different cancer cell lines (Raji, K562, Fen and HeLa). The result showed that these compounds had moderate-good cytotoxic activity. In addition, QSAR model shows the importance of N atom in cytotoxicity; Ca^2+^ channels.

## Introduction

The 1, 4-dihydropyridine (DHP) derivatives, known as calcium channel antagonists, are used for treatment of cardiovascular diseases such as hypertension and angina pectoris (1-4). It has been discovered that DHPs posses a wide range of other beneficial biological activities such as anticonvulsant analgesic and are also used as a chemical drug delivery systems especially in brain delivery (5-8). Recently, it has been proved that these compounds make a new class of multidrug resistance (MDR) reversals in cancer treatment. Therefore, extensive investigations were made in order to find new DHP derivatives as MDR reversal agents (9-11).

Beside the reversing activity of DHPs, there are several reports about their intrinsic Cytotoxicity. Particularly, some derivatives showed significant cytotoxicity such as dexniguldipine and some dibenzoyl derivatives (12-15). In addition, there are some reports on the effects of DHPs on potentiation of antitumoral and antimetastatic activity of some common cytotoxic drugs (16). Mechanistic investigations were proved that cytotoxicity of DHPs may not be the result of interaction with Ca^2+ ^channels although it might be related to other pathways in which calcium is involved such as calcium/calmodulin pathway or other cellular pathways like inhibition of topoisomerase I (12, 17, 18).

In this study, we performed an investigation about the intrinsic cytotoxicity of some derivatives of DHP which were bearing a nitroimidazole group on the C_4 _position.

## Experimental

*Dihydropyridine compounds*

All compounds used in this study have been synthesized in our chemistry lab and their synthesis process and their Ca^+2^ blocking activity were previously reported (8, 19-23). Structures of compounds and their Ca channel blocking activity are presented in Table 1.

**Table 1:** Structure of DHP derivatives used in this study and their calcium channel antagonist activity.

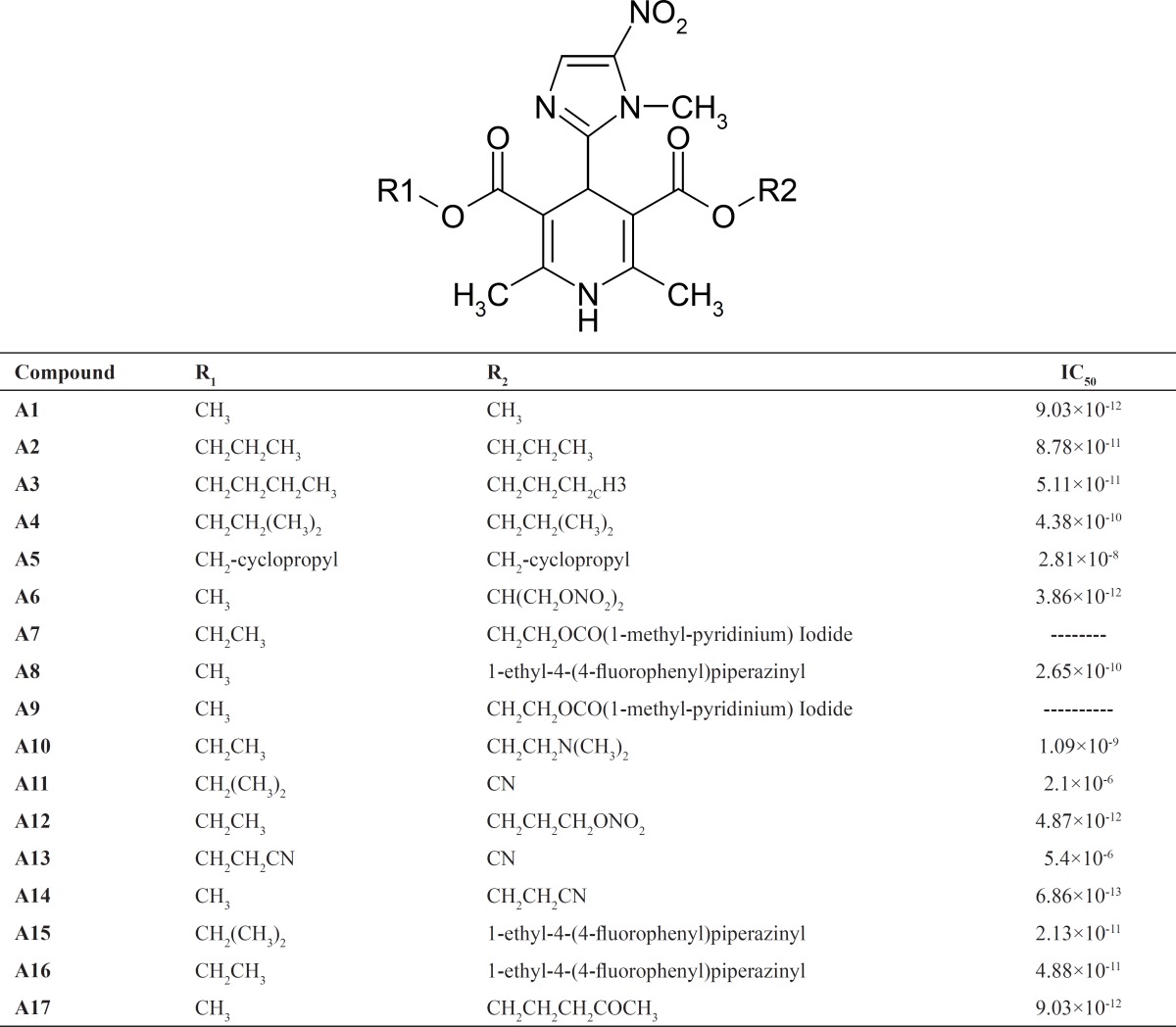

*Cell lines and reagents*

Five cell lines including K-562 (chronic myelogenous leukemia), HeLa (carcinoma of the cervix), Raji (Human, B cell Lymphoma), and Fen (bladder carcinoma cell line) were all obtained from the Iran Pasteur Institute, Tehran, Iran. Cells were cultured in RPMI-1640 (Sigma, USA) supplemented with 10% fetal bovine serum (Gibco, USA), 100 IU/mL penicillin and 100 μg/mL streptomycin (Biosera, England). Cells were cultured in 50 cm^3^ flask (Nunc, Denmark) with 5 mL of culture medium in humidified 5% CO_2 _incubator at 37°C.

*Cytotoxicity evaluation*

Appropriate amount of compounds was mixed with DMSO (dimethyl sulfoxide). Then, four concentration (10, 100, 50, 1000 μM) were made with serial dilution by RPMI-1640. DMSO, as the negative control, was diluted with the same method. For positive control, doxorubicin was mixed with RPMI-1640 to make similar concentrations. MTT [3-(4, 5-dimethylthiazole-2-yl)-2, 5-diphenyl tetrazolium bromide] (Sigma, USA), was dissolved in PBS (phosphate buffered saline) at 5 mg/mL. Each well of the plate was filled with 1-5×10^4^ cells (depending on the cell line) in 90 μL cultured medium. Then, 10 μL from the stock solutions of compounds, negative controls and positive controls, was added as triplicate to the wells to reach the final concentration of 1, 10, 50, 100 μM. Three wells containing only the same number of cells left in each plate. Plates were kept in a humidified incubator for 48 h. After the incubation period, MTT assay was carried out by the procedure described by Jabbar *et al. *(24).

*QSAR studies*

Chemical structure of the molecules was built using HyperChem software (Version 7, Hypercube Inc., http://www.hyper.com, USA) for the structural chemistry. Gaussian98 was operated to optimize the molecular structure. The structures were optimized by Ab initio calculations at the level of RHF/ STO-3G. No molecular symmetry constraint was applied; rather full optimization of all bond lengths and angles was carried out. Highest occupied molecular orbital (HOMO) and lowest unoccupied molecular orbital (LUMO) energies, molecular polarizability (MP) and molecular dipole moment (MDP) were all calculated by Gaussian98. Local charges (LC) at each atom were calculated according to both Mulliken population and natural population analysis methods. Some theoretical QSAR descriptors including physicochemical properties and topological indices were calculated using HyperChem (Hypercube Inc.) and DRAGON (http://www.talete.mi.it/ dragon_exp.htm, Italia) software. The MLR analysis was employed to derive the QSAR models for different cytotoxic activities. In order to make better QSAR models, IC_50 _of compounds was transformed into the logarithmic scale (log IC_50_). MLR and correlation analyses were carried out by the statistics software SPSS 11.5 version. Before any MLR analysis, the correlation between the selected descriptors was examined (Table 3) and collinear descriptors (r > 0.90) were determined. Among these descriptors, the one which had higher correlation with the dependent variable was retained and the others were removed from the descriptor data matrix. The remaining descriptors were used to construct the MLR model, in accordance with the stepwise selection and elimination method. The better regression models were selected on the basis of the higher R, f-value (a statistic of assessing the overall significance) and the lower SEE. Cross-validation procedure [leave-one-out (Q^2^_LOO_)] was applied to measure the predictive capabilities of the models by using MATLAB 6.5 program (25).

## Results and Discussion

The data of cytotoxicity is shown in Tables 2, 3 and 4. IC_15_, IC_30_ and IC_50 _were calculated for each compound. The most cytotoxic effect was seen on Raji cell line while the lowest activity was observed in K562 and HeLa, since at least 7 compounds had IC_30_ and IC_50_ of higher than 100 μM.

**Table 2:** Cell growth inhibitory activity of compounds a-e *in-vitro *(IC_15_

**IC**_15_^a^ **(μM)**					
HeLa	Fen	K562	Raji	Compound
38.1	< 1	41.8	< 1	
54.7	< 1	84.0	< 1	**A2**
38.8	< 1	6.2	< 1	**A3**
45.5	< 1	20.2	< 1	**A4**
72.4	< 1	< 1	< 1	**A5**
< 1	< 1	< 1	< 1	**A6**
< 1	< 1	4.1	< 1	**A7**
1.2	< 1	< 1	< 1	**A8**
< 1	< 1	< 1	< 1	**A9**
< 1	< 1	< 1	< 1	**A10**
53.5	51.5	52.5	> 100	**A11**
< 1	< 1	< 1	< 1	**A12**
< 1	< 1	< 1	< 1	**A13**
< 1	< 1	< 1	< 1	**A14**
< 1	< 1	< 1	< 1	**A15**
< 1	< 1	< 1	< 1	**A16**
35.5	44.7	44.4	22.4	**A17**
< 1	< 1	< 1	< 1	**Doxorubicin**
81.1	71.4	80.4	92.4	**DMSO**

a: IC_15_ is the molar concentration causing 15% growth inhibition of tumor cells

**Table 3:** Cell growth inhibitory activity of compounds a-e *in-vitro *(IC_30_).

**IC**_30_^a^ **(μM)**				**Compound**
		
**HeLa**	**Fen**	**K562**	**Raji**		
100 <	33.1	100 <	58.3	**A1**
100 <	57.8	100 <	1 >	**A2**
100 <	37.3	100 <	32.5	**A3**
100 <	58.9	100 <	1 >	**A4**
100<	71.1	100 <	100<	**A5**
1 >	1 >	1 >	1 >	**A6**
12.6	10.5	100 <	12.3	**A7**
5.6	1.4	1.3	1.4	**A8**
5.4	1.1	1.1	1.1	**A9**
3.1	1 >	1 >	1	**A10**
100 <	100 <	100 <	100 <	**A11**
1.7	1.2	1.2	1.4	**A12**
1 >	1 >	1 >	1 >	**A13**
1 >	1 >	1 >	1 >	**A14**
3.1	1.3	2.1	1.1	**A15**
3.4	1>	1 >	1	**A16**
100 <	100 <	1 >	100<	**A17**
1 >	1 >	1 >	1 >	**Doxorubicin**
100 <	100 <	100 <	100 <	**DMSO**

a: IC_30_ is the molar concentration causing 30% growth inhibition of tumor cells

**Table 4:** Cell growth inhibitory activity of compounds a-e *in-vitro *(IC_50_).

**IC**_50_^a^ **(μM)**				
	
**HeLa**	**Fen**	**K562**	**Raji**	**Compound**
> 100	> 100	> 100	> 100	**A1**
> 100	> 100	> 100	> 100	**A2**
> 100	> 100	> 100	> 100	**A3**
> 100	> 100	> 100	> 100	**A4**
> 100	> 100	> 100	> 100	**A5**
5.4	4.1	2.8	4.0	**A6**
68.3	56.3	> 100	69.3	**A7**
30.9	15.1	14.1	15.4	**A8**
29.4	6.8	3.0	7.1	**A9**
25.3	4.3	3.8	8.1	**A10**
> 100	> 100	> 100	> 100	**A11**
14.9	14.3	14.3	14.1	**A12**
< 1	< 1	< 1	< 1	**A13**
2.8	2.4	2.5	3.1	**A14**
23.4	8.8	20.2	8.4	**A15**
28.5	7.2	3.3	8.2	**A16**
> 100	> 100	> 100	> 100	**A17**
< 1	< 1	< 1	< 1	**Doxorubicin**
> 100	> 100	> 100	> 100	**DMSO**

a: IC_50_ is the molar concentration causing 50% growth inhibition of tumor cells.

However, synthesized compounds had moderate cytotoxicity. It was obvious that symmetric compounds are significantly less potent since all of them have IC_50_ greater than 100 μM. On the other hand, the asymmetric derivatives had good cytotoxicity. Among them, compound A13 showed the best activity since its IC_50_ in all cell lines were lower than 1 μM which was comparable with the reference drug (doxorubicin). 

*QSAR studies*

MLR analysis with the stepwise selection and elimination of variables was utilized to model the structure-activity relationships with diverse set of descriptors. 

In the first step, it was tried to find an appropriate model for cytotoxic activity of all compounds in Raji. The obtained equation is as follows: 

1og *IC*_50 _= 5.966 - 0.551 (±0.097) *nN *- 0.897 (±0.242) C - 040

N = 17, *R*_2 _= 0.812, *Q*^2^_LOO_ = 0.772, *F *= 30.1, *SE *= 0.311

In this equation, the values in the parenthesis stand for the standard deviation of the coefficients. N, R^2^, SE, and F are number of components, correlation coefficient, standard error of regression, and Fisher’s F-ratio, respectively. Noticeably, this equation has a good statistical feature, which can explain and predict 82% of variances in the cytotoxic activity data. Q^2^_LOO_ represents acceptable correlation coefficient, which is close to that of calibration. 

This explains the satisfactory prediction ability and stability of the resulted model. This two-parametric equation, holding the number of nitrogen atoms (nN) and an atom center fragment parameters (C-040), implies the importance of constitutional parameters in their activity. 

The next equation was obtained for cytotoxicity of K562 cell line:

1og *IC*_50_ = 6.002 - 0.571 (±0.142) *nN *- 0.878 (±0.353)

*C *- 040

N = 17, *R*_2_ = 0.680, *Q*^2^_Loo_ = 0.600, *F *= 14.88, *SE *= 0.453

It is obvious that cytotoxic activity K562 cell line has a great similarity with the activity in Raji cell line, though statistical quality of latter equation is much lower than the first equation. This might imply the similar mechanism of cytotoxicity in both cell lines.

The next equation was obtained for the cytotoxicity of Fen cell line: 

1og *IC*_50_ = 4.147 - 0.689 (±0.117) *nN *- 0.367 (±0.140) *MLOGP*

N = 17, R_2_ = 0.812, *Q*^2^_LOO_ = 0.636, *F *= 19.1, *SE *= 0.383

In this equation as well, the number of nitrogen is the most important factor while, dissimilar to the former equation, the second parameter is MLOGP which octanol-water partition coefficient. This may suggest that transversing of biological membranes is a key factor in cytotoxic activity in this cell lines, since it was proved that log P is a crucial factor in crossing the biological membrane (26).

Finally, an equation for HeLa cell line was obtained as described below:

1og *IC*_50 _= 7.23 - 1.641 (±0.290) *nCN *- 0.211 (±0.036) *SLC*DHP *+ *9.123 (±2.91) *nHAcc*

N = 17, *R*_2_ = 0.856, *Q*^2^_LOO_ = 0.644, *F *= 25.8, *SE *= 0.257

This three-parametric equation containing the holding number of nitrile groups (nCN), sum of local charge on DHP ring (SLC_DHP_) and the number of acceptor atoms for hydrogen bonding (nHAcc), shows that cytotoxic activity of DHP derivatives in this cell line probably is slightly different from other cell lines, while the presence of nitrogen (here in nitrile form) is a key factor. 

Overall, it can be concluded that presence of nitrogen has a crucial importance in the cytotoxicity DHP derivatives in both total number of nitrogen and also groups containing nitrogen (*i.e. *nitrile group). 

On the other hand, modeling the Ca^+2^ channel blocking activity of these compounds gives the following equation:

1og *IC*_50_ = 9.74 + 3.60 (±0.60) *SLC*NI - 1.562 (±0.352)

*H *- 046 - 1.459 (±0.569)*Mv*

*N *= 15, *R*_2_ = 0.852, *Q*^2^_LOO_ = 0.701, *F *= 19.1, *SE *= 0.49

Where SLC_NI_ is the sum of local charge on nitroimidazole ring, H-046 is an atom-centered fragment (H attached to C0 (sp3)) and Mv is the mean atomic van der Waals volume. This equation shows the importance of charges in C4 position while volume of molecules has negative effect on Ca^+2^ channel blocking activity. 

## Conclusion

DHPs are one of the major classes of Ca^2+^ channel blockers. Recently, their MDR reversing activity attracted many researchers. In this way, a large set of new derivatives of DHP were synthesized as MDR reversals, some of which have reached to the upper stage of studies. 

In addition, some derivatives showed intrinsic cytotoxicity on cancer cell lines. As a remarkable example, dexniguldipine, a potent MDR reversal, exhibited strong cytotoxicity on various cell lines. On the other hand, it was shown that nitroimidazole and its derivatives possessed appropriate cytotoxic properties (27-29). Therefore, it seems that DHP compounds including nitroimidazole moiety at C4 position may have strong cytotoxicity due to the simultaneous presence of DHP base structure and nitroimidazole substituent. In the present study, cytotoxic activity of some DHP derivatives was evaluated on four different cancer cell lines. Asymmetric derivatives showed good cytotoxicity while symmetric ones were less potent. 

In addition, QSAR studies revealed the significance of N atom and group containing it in cytotoxic activity. This is compatible with previous findings about MDR reversing activity of DHPs which demonstrated the good association of amine groups with MDR reversing effect. For example, Ford’s findings showed that tertiary and cyclic amine groups are decisive for MDR reversing activity (30) and in recent years, in most synthesized DHP derivatives as MDR modulators, this rule has been applied (10, 31). This correlation between MDR reversing activity and cytotoxic effect might be the sign of possible similar mechanisms interfering in both phenomena.

On the other hand, QSAR model of Ca^+2^ channel blocking activity confirms that key factors in this activity are to some extent different from cytotoxic activity. For example, while local charges of DHP is an important feature in cytotoxic effect, the significant part for Ca^+2^ channel blocking activity is nitroimidazole moiety. Besides, the most significant factor in cytotoxic effect (number of nitrogen atoms) does not have any considerable role in Ca^+2^ channel blocking activity. Another remarkable point about Ca^+2^ channel blocking activity is that the existence of local charge of nitroimidazole moiety in QSAR model is in agreement with previous works which highlighted the importance of the substituent of C4 position (32).
